# Supplementary material for: Bacterial age distribution in soil – Generational gaps in adjacent hot and cold spots
Source: PLoS Comput Biol. 2022 Feb 25;18(2):e1009857. doi: 10.1371/journal.pcbi.1009857 (PMC8906644; doi:10.1371/journal.pcbi.1009857)
Supplement: S1 Fig — (PDF) [file pcbi.1009857.s001.pdf]

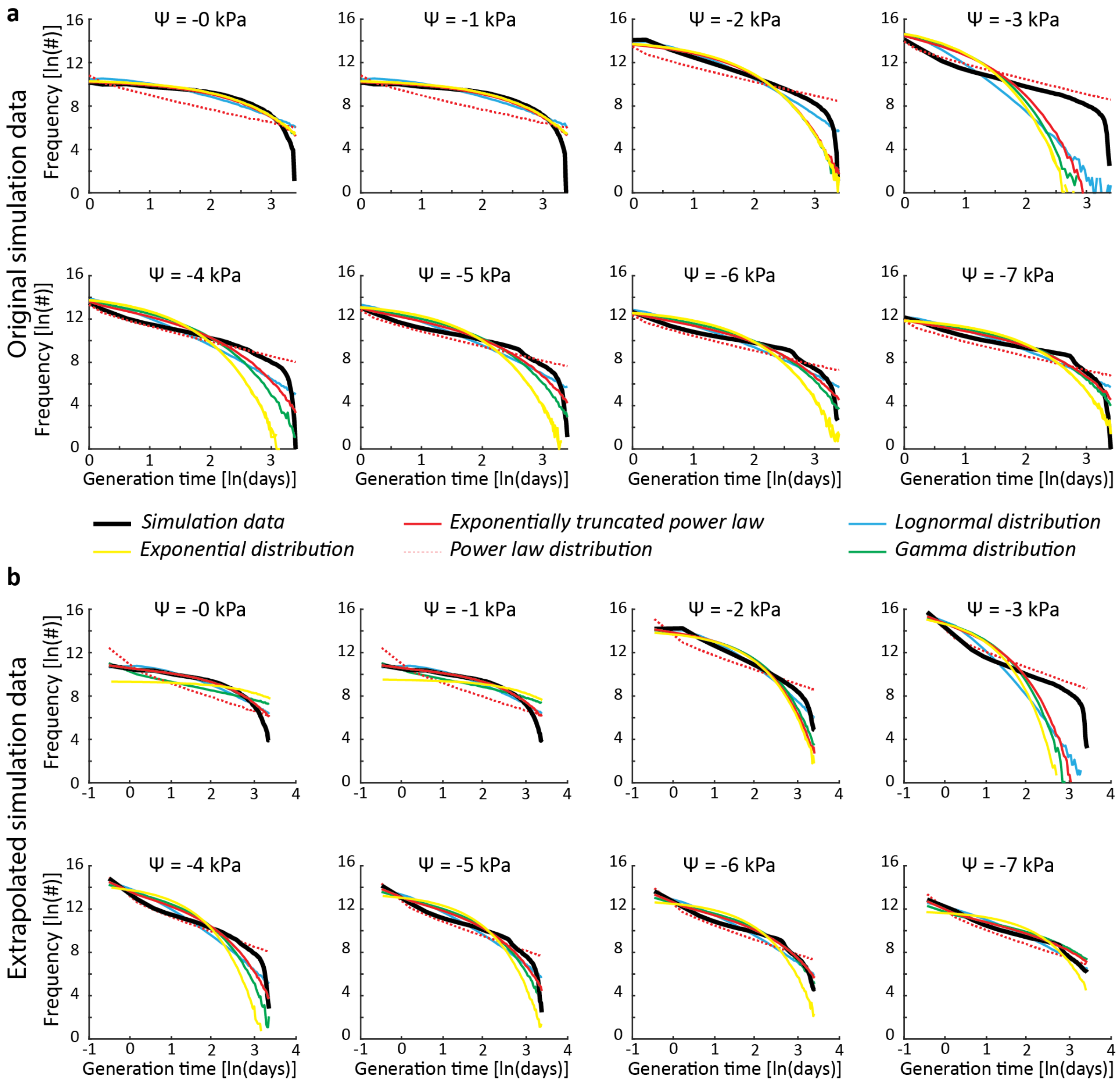

S1 Figure: Long tailed distributions fitted to the IndiMeSH simulation data using maximum likelihood estimation. a) Five long-tailed distributions fitted to the original simulation data using maximum likelihood estimation for all hydration conditions. b) Five long-tailed distributions fitted to the extrapolated simulation data using maximum likelihood estimation for all hydration conditions.
