# Supplementary material for: Bacterial age distribution in soil – Generational gaps in adjacent hot and cold spots
Source: PLoS Comput Biol. 2022 Feb 25;18(2):e1009857. doi: 10.1371/journal.pcbi.1009857 (PMC8906644; doi:10.1371/journal.pcbi.1009857)
Supplement: S2 Fig — (PDF) [file pcbi.1009857.s002.pdf]

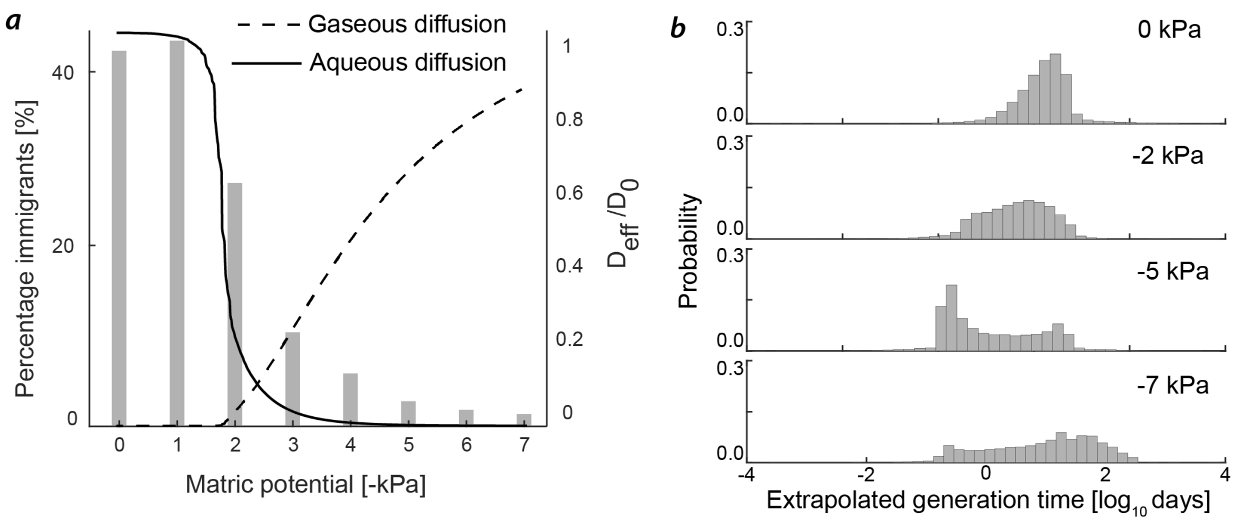

S2 Figure: Cell dispersal and diffusion regime determine bacterial generation time distribution. a) Wet conditions enable cell dispersal and facilitate immigration of individual lineages towards the carbon source whilst restricting oxygen diffusion. Immigration is defined as a lineage proliferating close to the carbon source that was originally inoculated more than half of the simulated radius of the domain away (i.e. was required to travel at least 5 mm). Dry conditions suppress bacterial motility due to pinning forces but enable ample oxygen diffusion through the gaseous phase. b) The ambient soil conditions and characteristics shape the bacterial generation time distribution in which a bimodal generation time distribution emerges under drier soil conditions (due to unhindered rapid growth in hotspots and progressively slower growth in cold spots).
