# Supplementary material for: Bacterial age distribution in soil – Generational gaps in adjacent hot and cold spots
Source: PLoS Comput Biol. 2022 Feb 25;18(2):e1009857. doi: 10.1371/journal.pcbi.1009857 (PMC8906644; doi:10.1371/journal.pcbi.1009857)
Supplement: S3 Fig — (PDF) [file pcbi.1009857.s003.pdf]

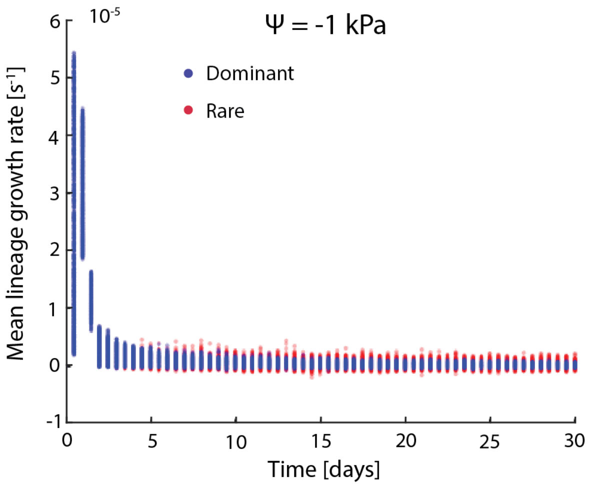

S3 Figure: Mean growth rates of individual lineages through time for two contrasting hydration conditions. Motile behavior in wet conditions (-1 kPa) results in a homogeneous growth rate of all individual lineages (both dominant and rare, with rare primarily being immigrants to the hotspot). Dry conditions in contrast follow a similar pattern only in the initial stages of the simulation and later show a wide distribution of growth rates.
