# Supplementary material for: Bacterial age distribution in soil – Generational gaps in adjacent hot and cold spots
Source: PLoS Comput Biol. 2022 Feb 25;18(2):e1009857. doi: 10.1371/journal.pcbi.1009857 (PMC8906644; doi:10.1371/journal.pcbi.1009857)
Supplement: S4 Fig — (PDF) [file pcbi.1009857.s004.pdf]

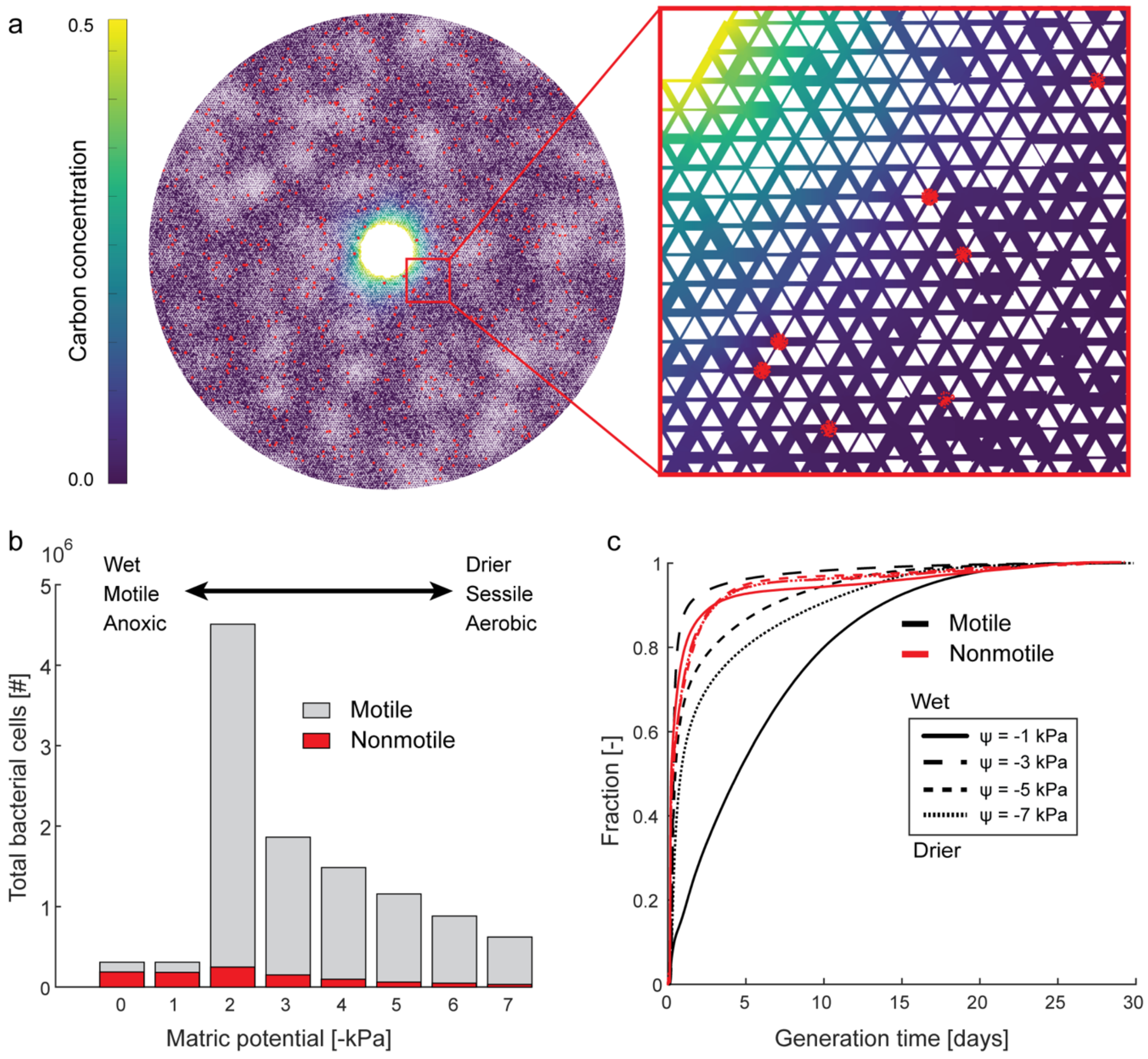

S4 Figure: Influence of cell motility on bacterial proliferation and generation time distributions. a) Spatial distribution of bacterial cells in the soil microbial hotspot domain that are clustered at the inoculation point. Considering non-motile bacterial cells, small sessile colonies form around each inoculated cell that results in high localized competition for resources. Since these colonies are sparsely distributed (i.e. overall low cell density in the domain), they do not sufficiently alter or self-engineer (i.e. consume carbon or oxygen) the environments around the hotspots, subsequently, the system is governed primarily by the diffusive flux of carbon through the aqueous phase. b) This diffusive limitation results in overall population sizes that are an order of magnitude smaller when compared to motile bacterial species. c) The diffusive limitations also induced homogenization of the generation time distribution for these species where under all hydration conditions, very similar generation time distributions were realized with the exception of saturated conditions in which the cells closest to the carbon source are additionally oxygen limited and thus have a heavier tail of the distribution.
