# Supplementary material for: Bacterial age distribution in soil – Generational gaps in adjacent hot and cold spots
Source: PLoS Comput Biol. 2022 Feb 25;18(2):e1009857. doi: 10.1371/journal.pcbi.1009857 (PMC8906644; doi:10.1371/journal.pcbi.1009857)
Supplement: S5 Fig — (PDF) [file pcbi.1009857.s005.pdf]

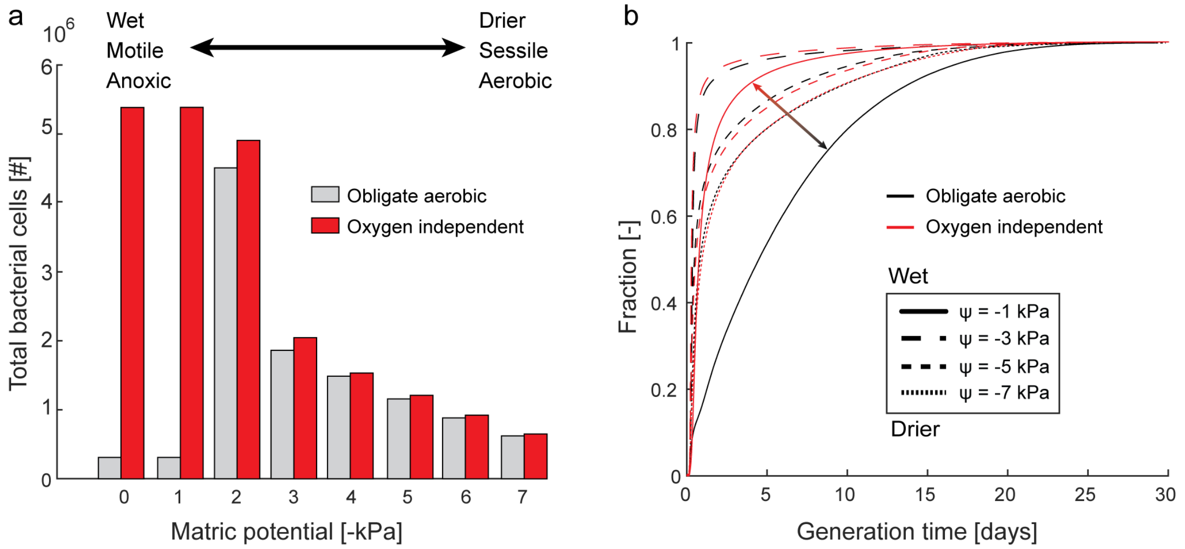

S5 Figure: Comparison of simulation between obligate aerobic and oxygen independent (facultative anaerobic) species. a) In comparison to the obligate aerobic species, removing the oxygen limitation results in rapid proliferation of the bacterial population in saturated conditions (since they are not limited by the slow diffusion of oxygen through the saturated pores) and generally a slightly larger population size. All other terms (growth rate, maintenance and yield) were kept congruent to the obligate aerobic species for sake of comparison. b) Overall, the generation time distributions are very similar to the obligate aerobic scenario for unsaturated conditions. In saturated conditions, the rapid proliferation of the cells results in a shift of the generation time distribution towards shorter generation times (as highlighted by the arrow). However, nutrient limitation due to rapid growth of all cells (relocation of the entire population towards the carbon rich center) results in longer average generation times when compared with intermediate hydration conditions (-3 kPa).
