# Supplementary material for: Bacterial age distribution in soil – Generational gaps in adjacent hot and cold spots
Source: PLoS Comput Biol. 2022 Feb 25;18(2):e1009857. doi: 10.1371/journal.pcbi.1009857 (PMC8906644; doi:10.1371/journal.pcbi.1009857)
Supplement: S6 Fig — (PDF) [file pcbi.1009857.s006.pdf]

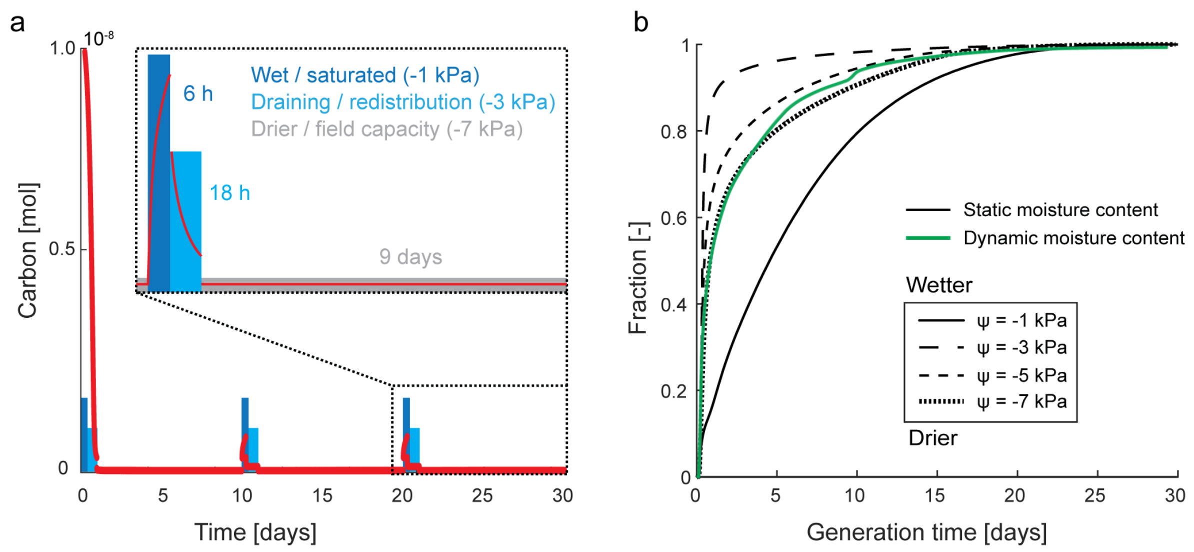

S6 Figure: Dynamic hydration conditions and resulting generation time distribution. a) We simulate three rain events in a thirty-day period by including dynamic variations of the moisture content. Each rain event constitutes of 6h saturated soil conditions (-1 kPa) followed by 18h of intermediate moisture conditions during which the aqueous phase redistributes (-3 kPa) and drier conditions at the characteristic hydration condition of the soil (-7 kPa) until the next rain event. Each rain event is characterized by an influx of carbon due to the higher aqueous diffusion fluxes within saturated pores. The inset shows details of a single cycle with arbitrary units for both carbon dynamics and hydration conditions. b) Dynamic conditions result in a slight shift of the overall generation distribution towards shorter generation times. This is due to the relocation of individual times during saturated conditions towards more favorable conditions, essentially acting to erode the tail of the distribution.
