# Supplementary material for: Bacterial age distribution in soil – Generational gaps in adjacent hot and cold spots
Source: PLoS Comput Biol. 2022 Feb 25;18(2):e1009857. doi: 10.1371/journal.pcbi.1009857 (PMC8906644; doi:10.1371/journal.pcbi.1009857)
Supplement: S1 Table — (PDF) [file pcbi.1009857.s007.pdf]

S1 Table: Experimental details of data used in Fig 1.

Authors + Year	***Sample***	***Method***	***Conditions***	***Ref***
Barber & Lynch, 1977	Rhizosphere	Helber Counting Chamber	Growth rates during different stages of root development	[1]
Söderberg & Bååth, 1998	Rhizosphere of barley	Thymidine and leucine incorporation	Looked at the location on the root	[2]
Bååth & Johansson, 1990	Rhizosphere of *Brassica*	Thymidine and leucine incorporation	Growth rates in rhizoplanes of plants with different ages	[3]
Olsson, S., Bååth, E. & Söderström, 1987	Rhizosphere	Agarose plate counting	Added microsclerotia at different concentrations	[4]
Bowen & Rovira, 1976	Rhizosphere and bulk soil	Agarose plate counting	Only rhizosphere data used in our study	[5]
Bennett & Lynch, 1981	Rhizosphere of gnotobiotic plants	Agarose plate counting	Looked at rhizosphere of different plants	[6]
Christensen, Funck-Jensen & Kjøller, 1989	Rhizosphere of sugar beet	Thymidine and leucine incorporation	Single bacterial strain in sterilized plant rhizosphere	[7]
Bloem et al., 1992	Bulk soil	Cell counts, FDDC	Investigated the influence of hydration conditions	[8]

1. Barber DA, Lynch JM. Microbial growth in the rhizosphere. Soil Biol Biochem. 1977;9: 305–308. doi:10.1016/0038-0717(77)90001-3

2. Söderberg KH, Bååth E. Bacterial activity along a young barley root measured by the thymidine and leucine incorporation techniques. Soil Biol Biochem. 1998;30: 1259–1268. doi:10.1016/S0038-0717(98)00058-3

3. Bååth E, Johansson T. Measurement of bacterial growth rates on the rhizoplane using 3H-thymidine incorporation into DNA. Plant Soil. 1990;126: 133–139. doi:10.1007/BF00041379

4. Olsson S, Bååth E, Söderström B. Growth of Verticillium dahliae Kleb. hyphae and of bacteria along the roots of rape (Brassica napus L.) seedlings. Can J Microbiol. 1987;33: 916–919. doi:10.1139/m87-159

5. Bowen GD, Rovira AD. Microbial colonization of plant roots. Annu Rev Phytopathol. 1976;14: 121–144. doi:10.1146/annurev.py.14.090176.001005

6. Bennett RA, Lynch JM. Bacterial growth and development in the rhizosphere ofgnotobiotic cereal plants. Microbiology. 1981;125: 95–102. doi:10.1099/00221287-125-1-95

7. Christensen H, Funck-Jensen D, Kjøller A. Growth rate of rhizosphere bacteria measured directly by the tritiated thymidine incorporation technique. Soil Biol Biochem. 1989;21: 113–117. doi:10.1016/0038-0717(89)90019-9

8. Bloem J, de Ruiter PC, Koopman GJ, Lebbink G, Brussaard L. Microbial numbers and activity in dried and rewetted arable soil under integrated and conventional management. Soil Biol Biochem. 1992;27: 655–665. doi:10.1016/0038-0717(92)90044-X
