# Supplementary material for: Bacterial age distribution in soil – Generational gaps in adjacent hot and cold spots
Source: PLoS Comput Biol. 2022 Feb 25;18(2):e1009857. doi: 10.1371/journal.pcbi.1009857 (PMC8906644; doi:10.1371/journal.pcbi.1009857)
Supplement: S2 Table — (PDF) [file pcbi.1009857.s008.pdf]

S2 Table: Parameters used in the mathematical model (IndiMeSH).

Parameter	Description	Value	Unit	Reference
D_{Carbon}	Diffusion coefficient of carbon	$5.9*10^{-10}$	m^2s^{-1}	**[1]**
D_{O2}	Diffusion coefficient of oxygen	$2*10^{-9}$	m^2s^{-1}	**[2]**
m_0	Average bacterial mass	$1*10^{-15}$	kg	**[3]**
m_{max}	Mass at division	$2*m_0/1.433$	kg	**[4]**
m_{crit}	Mass at cell death	$0.2*\ m_{max}$	kg	**[4]**
Y	Cell yield	0.06	kg mol^{-1} Carbon	**[5]**
μ_{max}	Maximum growth rate	$6.9*10^{-6}$	s^{-1}	**[6]**
$K_{C,lim}$	Monod half saturation coefficient for carbon	0.05	mM	**[5]**
$K_{O2,lim}$	Monod half saturation coefficient for oxygen	0.0063	mM	**[7]**
p_0	Unbiased tumbling probability	0.25	s^{-1}	**[8]**
χ	Chemotactic sensitivity	$2*10^{-8}$	m^2s^{-1}	**[9]**
v	Corrected cell velocity for 1-D movement	2	ms^{-1}	**[8]**

1. Southard MZ, Dias LJ, Himmelstein KJ, Stella VJ. Experimental determinations of diffusion coefficients in dilute aqueous solution using the method of hydrodynamic stability. Pharm Res. 1991;8: 1489–1494.

2. Han P, Bartels DM. Temperature Dependence of Oxygen Diffusion in H2O and D2O. J Phys Chem. 1996;100: 5597–5602. doi:10.1021/jp952903y

3. Phillips R, Kondev J, Theriot J, Garcia H, Chasan B. Physical Biology of the Cell. Am J Phys. 2010;78: 1230. doi:10.1119/1.3459039

4. Kreft JU, Booth G, Wimpenny JWT. BacSim, a simulator for individual-based modelling of bacterial colony growth. Microbiology. 1998;144: 3275–3287. doi:10.1099/00221287-144-12-3275

5. Kornaros M, Zafiri C, Lyberatos G. Kinetics of denitrification by *Pseudomonas denitrificans* under growth conditions limited by carbon and/or nitrate or nitrite. Water Environ Res. 1996;68: 934–945. doi:10.2175/106143096X127947

6. Lardon LA, Merkey B V., Martins S, Dötsch A, Picioreanu C, Kreft JU, et al. iDynoMiCS: Next-generation individual-based modelling of biofilms. Environ Microbiol. 2011;13: 2416–2434. doi:10.1111/j.1462-2920.2011.02414.x

7. Calderer M, Jubany I, Pérez R, Martí V, de Pablo J. Modelling enhanced groundwater denitrification in batch micrococosm tests. Chem Eng J. 2010;165: 2–9. doi:10.1016/j.cej.2010.08.042

8. Berg HC, Brown DA. Chemotaxis in *Escherichia coli* analysed by three-dimensional tracking. Nature. 1972;239: 500–504. doi:10.1038/239500a0

9. Kim M, Or D. Individual-Based Model of Microbial Life on Hydrated Rough Soil Surfaces. PloS One. 2016;11: e0147394. doi:10.1371/journal.pone.0147394
