# Supplementary material for: Bacterial age distribution in soil – Generational gaps in adjacent hot and cold spots
Source: PLoS Comput Biol. 2022 Feb 25;18(2):e1009857. doi: 10.1371/journal.pcbi.1009857 (PMC8906644; doi:10.1371/journal.pcbi.1009857)
Supplement: S3 Table — (PDF) [file pcbi.1009857.s009.pdf]

S3 Table: Simulated mean cell age [days] as a function of matric potential for different cumulative biomass cutoff threshold. The mean cell age for the abundant biomass is calculated from the most abundant bacterial cell lineages up to a percentage of the total biomass in the simulation domain (cumulative biomass up to the cutoff value). The sparse biomass constitutes the remaining cell lineages. The mean cell age of the abundant fraction is evidently a function of hydration conditions whereas the chosen threshold does not influence the result significantly. The mean cell age of the rare fraction on the other hand is significantly altered by the choice of cumulative cutoff value in addition to prevailing hydration conditions.

Matric potential [-kPa]		*Cumulative biomass cutoff threshold*		
		**0.9**	**0.99**	**0.999**
Dominant	**0**	9.1	9.2	9.2
	**1**	9.1	9.2	9.2
	**2**	7.6	7.6	7.6
	**3**	2.7	2.7	2.7
	**4**	3.9	3.9	3.9
	**5**	4.8	4.8	4.8
	**6**	5.3	5.4	5.4
	**7**	6.4	6.4	6.5
Rare	**0**	9.6	11.0	12.4
	**1**	9.6	11.1	12.6
	**2**	7.7	8.4	11.8
	**3**	2.9	3.8	11.9
	**4**	4.0	5.0	13.3
	**5**	4.9	6.1	16.7
	**6**	5.5	6.9	20.5
	**7**	6.7	8.7	26.0
