# Supplementary material for: Bacterial age distribution in soil – Generational gaps in adjacent hot and cold spots
Source: PLoS Comput Biol. 2022 Feb 25;18(2):e1009857. doi: 10.1371/journal.pcbi.1009857 (PMC8906644; doi:10.1371/journal.pcbi.1009857)
Supplement: Attachment — Submitted filename: PCOMPBIOL_D_21_00669_Review.pdf [file pcbi.1009857.s011.pdf]

Review of Manuscript PCOMPBIOL-D-21-00669

Review Summary

In this paper the authors generate and study the distribution of generation times in communities of bacterial species in local patches of soil. To this end, they use a previously-published spatially-explicit simulation model (Borer et al., 2019) that emulates networks of micropores and the hydrodynamics therein that govern the physical transport and exchange of gases, nutrients and microbial cells, as well as microbial metabolite exchanges and population growth. They find that the simulations yield relatively long-tailed (but not power-law) distributions of generation times across isogenic species populations, with the specific shape varying with hydration level (drier $\implies$ more power-law like). The authors derive a stochastic (mixture) model to support their hypothesis that real-world bacterial generation time distributions are likely to follow power-laws, and suggest that the disparity between this analytic model and the simulations is driven by the greater realism of the simulation model.

We found the line of investigation of this study very interesting, and the manuscript is well written and easy to follow. However, we feel that there is a lot going on "under the hood" that needs clarification. In particular, the simulation results here rely heavily on the previously-published model (Borer et al., 2019) and its specific parameterisations, forcing the reader to read *that* paper which still leaves a few key questions unanswered (more details below). We also think that the empirical data aggregation from the literature not as comprehensive as it could be, and have some concerns about the analytic derivation of the power-law distribution of generation times.

Overall, we think that this paper uncovers something of value in terms of the relative effects of hydration regimes on generation time distributions at small/local spatial scales. But in terms of absolute effects and generation time distributions in the "real" world, we find the key claims tenuous for the reasons detailed below. If the authors instead focus on addressing the issue of hot/cold spots themselves changing over time, and restrict themselves to discussing the implications of generation time differences that their model actually resolves (i.e., in the order of tens of days), then this paper would still be a valuable contribution (with the necessary caveats about the model's limitations). This would still allow them to engage in the discussion of side-by-side coexistence of slow generation-time microbes acting as "keepers of traits" and fast generation-time microbes undergoing rapid evolution at their maximal growth rate.

Our specific comments follow.

Major comments

1. **The simulation model**. There very little explanation of the (previously-published) model (Borer et al., 2019) used to generate the results of this paper. This makes it hard to understand the mechanistic basis as well as robustness (in the sense of parametrisation-dependence) of the presented results, and assess what impact the assumptions inherent in the underlying model could have on the final results. Four key questions in this regard that arise for us are:

   (a) Motility: how dependent are the results on the choice of the motility model and parameterisations? For example, the parameter definitions given in Table S1 (Lines 561–563) suggest that the mode of microbial motility considered is run and tumble movement + chemotaxis, something that is mentioned nowhere else in this manuscript, and ultimately one has to go back to the previous paper, and upon reading that, one is still not sure about the robustness of the specific parameterisations chosen.

   (b) There is no mention of species interactions in the local domains. The rGEM-based modelling of microbial cell growth and division presumably yields interactions between (and within) microbial species, but this issue is not addressed at all in the paper. It may well be that interactions

are moot here, but its unclear currently.

(c) How many species / isogenic strains were introduced into a patch? Were the 1000 cells "inoculated homogeneously across the domain" per species, or across all species? Are extinctions possible in this system, and how are they handled?

(d) As the focus here is on complex communities, is the model able to produce any elements of realistic macroecological patterns (e.g., a "canonical" species abundance distribution) seen in (soil or otherwise) microbial communities (Grilli, 2020)? Maybe this last point is a bit demanding because the published model has been validated to some degree in the previous paper, but these are complex dynamical systems, and some consideration/discussion of this is warranted.

2. **The empirical data and associated issues**. We agree that practically no studies have measured community-level generation times including variation within isogenic (species) lineage level variation. However, in our view these data can still be used to partially test the simulated model's predictions. Specifically, the central tendencies (means or medians) of the distributions underlying a mixture distribution should follow some canonical distribution (possibly also a power-law in this case?). Therefore, it would be meaningful to compare the distribution of means or medians of species-specific generation times emerging from model-simulated data with those of real datasets. With reference to this, the authors should consider published datasets on maximum growth rates that include soil-derived taxa (Corkrey et al., 2016; Smith et al., 2019). These also account for temperature variation, which would allow for a more thorough analysis of the (temperature-corrected) distributions of generation times of *potential*, randomly constituted local bacterial communities. That is, this would offer a null or neutral model for what the generation time distribution of mean/median values in multi-species communities might look like. This may also lead to the derivation of the generation time distribution of a local, well mixed microbial community under idealized nutrient and substrate conditions, providing a theoretical benchmark to compare more realistically mixed communities against.

   In terms of the plot shown in Fig 1, the empirical data panel is not a relevant point of comparison to the panel based on the simulations below it, because as the authors themselves point out, the literature reports only mean values of single populations. It would therefore be helpful to show another plot of (a representative) distribution of population means of simulated data so that a direct comparison can be made with empirical data.

   Also, it is not clear if/how the empirical data has been selected to generate a representative sample of generation times (the data aggregation are not described at all in the methods or elsewhere). For example, what sort of method is the 4-year data point based on? Is it really comparable to the others? And although the authors suggest that empirical data are biased towards detecting younger cells, why are the generation times in the empirical data in Fig 1 actually much longer than the simulated data?

3. **Analytic derivation of the generation times distribution**. The relevance of these results to the question of microbial age distributions hinges on the discovery of a power law scaling of generation time, which suggests a finite probability of observing extremely large times between cell division events. The derived exponent ($\alpha = 2$) differs substantially from the range of exponents found across matric potentials ($\alpha = 1.2 - 1.35$). This deviation is mentioned briefly in the discussion (Lines 250–255), but further investigation is deferred to future studies. This is matched by a derivation that is short and lacking in detail (Lines 375–392). We think that clarifying the assumptions made in the steps of this derivation is necessary to make the results of this paper clearly understandable. Beyond this, the derivation appears to implicitly make the assumption (Eq. 3) that the distribution of growth rates (k) can be treated to be uniform, and so the integral can be performed without a

weighting function. In the supplementary text to (Şimşek and Kim, 2019) (cited in the manuscript) the impact of non-uniform distributions of growth rates is considered. We wonder if this can be used to explain either the substantial deviation of the exponents or the fact that exponentially truncated power laws appear to fit better than power laws in the majority of cases.

4. There is an additional issue: the time-scale of physical mixing of bacterial cells, nutrients, and carbon substrates is annual or shorter (through periodic or intermittent precipitation and inundation). That is, arguably, mixing happens frequently enough to make cold and hot spots ephemeral physical entities in the timescales relevant here. Therefore, the wet/dry patches that the authors define (corresponding to population-dynamically hot/cold spots) are not static, but must surely themselves evolve over, say, a period weeks or months at the longest. With these considerations, any shortcomings of the model apart, we feel that the claim that cells and lineages of the (extreme) age suggested by this work seem highly unlikely empirically, unless one is talking about much deeper zones in soil than what is the focus of the modelling.

5. Overall then, although the possibility of ancient lineages co-existing with younger ones is an intriguing one, the results of the current paper are far from convincing in that regard. Ultimately, fits to the power-law are weak, and the exponential declines across all regimes (including the driest ones) not given due regard. We are unable to judge how valid the method of extrapolating the distributions of generation times mentioned in lines 154–157 is.

Other comments

- What about the role of (across-species) scaling of metabolic rate with cell size in generating power law distributions of generation times (DeLong et al., 2010)? Size presumably plays a key role in motility as well. Maybe worth discussing as it provide some additional mechanistic understanding about the real world phenomenon even if size is not considered in the simulation model.

- Lines 12–14: The claims made early in the abstract seem too bold to be supported by the present results of the paper or the literature. Specifically, the claim in the abstract that "much older living microorganisms (than $\sim$ 4850 years) are likely to be found in most soils" does not appear to be supported by any literature cited in the paper.

- Lines 20–23: This part of the abstract is a bit vague and makes no mention of the specific mechanisms investigated.

- Lines 29–31: Something is wrong with the sentence here (appears to be a fragment).

- Line 43: What are these relic traits? And example or two would help.

- Lines 103–111: Hydrological regime is clearly key to the emergent patterns from the simulations, but only gets mentioned at the start of the Results section rather than in the Introduction (or Abstract). Also, much of this start of the Results seems like information that should be presented in the Methods rather than here.

- Lines 113–114: The results pertain specifically to obligately aerobic bacteria. This is briefly defended in the Methods section. Some discussion of how these results be effected if e.g., dominantly, or facultatively anaerobic bacteria were considered?

- Line 126: There isn't really enough information provided in these plots to assess how well the power-law fits the datasets. The details are in the Supp info, but the fact that the data pretty much never fit a strict power-law would be better off shown in the main text.

- Lines 183–184: Exactly what is meant by "Power law distributed cell ages induce a broad range of reproductive success" could be clarified a bit.
- Lines 189–190: The method used for the classification of lineages is fairly opaque – please explain/clarify further
- Lines 253–254: Reference is made to an "assumption-free mechanistic model", a model without assumptions ceases to be a model, in our opinion.
- Lines 289–303: A large number of possible countervailing mechanisms are mentioned here, and it's noted that the actual generation time distribution will be a balance between the mechanism studied in this manuscript and these counteracting mechanisms. Given the importance placed on these here and the manuscript's stated aim of determining the possible ages of soil microbes is that no way that the impact of counteracting mechanisms could have been considered even briefly in the Results?
- Line 305: Why is evolutionary rate a function of nutrient conditions? Is this entirely due to faster growth rate meaning more cell divisions and thus more opportunity for mutations?
- Lines 331–333: The conclusion that microbes can be practically any age seems too bold a conclusion given the current results.
- Eqn 1: Worth mentioning that this is for symmetric division and defending that assumption.
- Lines 531–538: The choice of line style and color makes the lines in Fig S1A and S2A hard to see.
- Fig S1, Line 535: a negative sign is missing before "3 kPa"
- Lines 552–559: A lot is expressed in this figure caption, whilst the mention of this figure in the main text is very brief. Some of the explanation given here would be more helpful if shifted to the main text.

References

Borer, B., Ataman, M., Hatzimanikatis, V., and Or, D. (2019). Modeling metabolic networks of individual bacterial agents in heterogeneous and dynamic soil habitats (IndiMeSH). *PLoS Comput. Biol.*, 15(6):e1007127.

Corkrey, R., McMeekin, T. A., Bowman, J. P., Ratkowsky, D. A., Olley, J., and Ross, T. (2016). The Biokinetic Spectrum for Temperature. *PLoS One*, 11(4):e0153343.

DeLong, J. P., Okie, J. G., Moses, M. E., Sibly, R. M., and Brown, J. H. (2010). Shifts in metabolic scaling, production, and efficiency across major evolutionary transitions of life. *Proc. Natl. Acad. Sci. U. S. A.*, 107(29):12941–12945.

Grilli, J. (2020). Macroecological laws describe variation and diversity in microbial communities. *Nat. Commun.*, 11(1):1–11.

Smith, T. P., Thomas, T. J. H., Garcia-Carreras, B., Sal, S., Yvon-Durocher, G., Bell, T., and Pawar, S. (2019). Community-level respiration of prokaryotic microbes may rise with global warming. *Nat. Commun.*, 10(1).

Şimşek, E. and Kim, M. (2019). Power-law tail in lag time distribution underlies bacterial persistence. *Proceedings of the National Academy of Sciences*, 116(36):17635–17640.

Samraat Pawar & Jacob Cook
Department of Life Sciences, Silwood Park
Imperial College London

June 11, 2021
