# Supplementary material for: Bacterial age distribution in soil – Generational gaps in adjacent hot and cold spots
Source: PLoS Comput Biol. 2022 Feb 25;18(2):e1009857. doi: 10.1371/journal.pcbi.1009857 (PMC8906644; doi:10.1371/journal.pcbi.1009857)
Supplement: Attachment — Submitted filename: Responses to Reviewer.docx [file pcbi.1009857.s012.docx]

Responses to Reviewer’s Questions

First, we thank the reviewers for the insightful and constructive comments that resulted in new insights and improvements to the manuscript. During the revision, we have made substantial changes to the structure of the manuscript to provide more information on how hydration conditions shape microbial life in the introduction and expanded the discussion to provide details how anaerobic metabolism may shape the generation time distributions and suggest an experimental system that may be used to track and quantify the influence of diverging bacterial growth rates on the emerging population structure and evolutionary forces. We have also made substantial changes to the manuscript concerning the derivation of the heuristic model and derived the equations using the assumption of a Gamma distribution for the generation time distribution as frequently reported in the literature and integrated the resulting equation numerically to assess the impact of heterogeneous growth rate distributions. We have also generalized the tone of the manuscript to talk about heavy-tailed distributions and not focus on power law distributions.

In the following, we provide a point-by-point response to reviewers’ comments. Answers by the authors are in blue whilst the original text of the reviewers is in black. All indication of lines are taken from the clean version of the manuscript (i.e. not track-change document).

Reviewer #1:

Overview:

This paper explores how growth rates of bacteria are affected by soil moisture using individual based modeling (IBM). The model, which has previously been described in published papers, simulates physical aspects of the soil environment at scales that are relevant to the physiology of cells in terms of motility, access to substrates, and oxygen availability. Simulation across a range of matric potentials reveals that total abundance is maximized at intermediate moisture contents. The paper then examines the distribution of generation times with respect to water content and reports that they follow a power-law distribution such that a few younger lineages (in “hot spots”) contribute most to total abundance, yet many lineages (in “cold spots”) are collectively rare, accounting for less than 3% of the population. The model is based on principles and reasonable assumptions that will make sense to people who think about microorganisms living in the soil environment. What I like most about the paper is that it makes some conceptual extensions about the life history, demographics, and longevity of populations that are not often considered by soil ecologists.

It seems that a number of other papers have been published using the IBM platform presented in this paper. On the one hand, this means that the approach itself is not especially novel, nor does it represent a particularly major methodological advancement. On the other hand, it means that the model has been vetted to some degree. Therefore, this evaluation tends to focus more on the application of the IBMs to the major finding, which is that the life span of bacterial cells is predicted to follow a power law distribution. The implications are that in a typical gram of soil, young and old cells may be found together.

Major comments:

--In Figure 1, the literature reported data has a longer tail, and the simulations seem to have a Poisson distribution. If growth reflects births, then one might expect a Poisson process giving rise to times between generation that follow an exponential distribution, which would have implications for the inferences that are being made. It’s not clear if other distributions were considered, but a more rigorous treatment along these lines is recommended.

The assumption that a Poisson process gives rise to an exponential distribution of inter-division times is very plausible – in the literature the Gamma distribution is often reported to be describe the generation time distribution for single populations and we use this assumption in the extended derivation of the heuristic model. In general, our manuscript focusses on how ubiquitous conditions in soil may promote the emergence of old bacteria and long tailed generation time distributions and how these are shaped by hydration conditions. Evidently, there are numerous processes that will shape the generation time distribution in soil, and the dynamic and patchy nature of soil as a microbial habitat will most likely result in no single mathematical equation adequately describing the true distribution for the whole community. When revising the manuscript, we extended the comparison between simulation data and various heavy-tailed distributions including an exponential distribution and changed Figure 3 in the manuscript (to include an exponentially truncated power law) and changed Figure S1 to include multiple heavy-tailed distributions (Figure below). Throughout the manuscript, we have rephrased sections to describe the resulting distributions in terms of “heavy-tailed” distributions without specifically referring to a mathematically defined distribution.

**Figure S1: Long tailed distributions fitted to the IndiMeSH simulation data using maximum likelihood estimation.** a) Five long-tailed distributions fitted to the original simulation data using maximum likelihood estimation for all hydration conditions. b) Five long-tailed distributions fitted to the extrapolated simulation data using maximum likelihood estimation for all hydration conditions.

--Evolution is invoked in various parts of the paper. For example, the abstract talks about “evolutionary milestones” and the “differential pace of evolution”. Unless I missed some critical details, there are no evolutionary processes in the IBMs, unless one wants to view motility as gene flow, but this is not explicitly developed. There is not genetic or phenotypic variation among individuals, that might be reflected by mutation, selection, or drift. While I think there are interesting implications for variation in growth rate for the evolution of populations, the model seems to be inadequate for making such statements. I’m concerned that a casual reader might be led to think and cite the paper based on claims that are not rigorously assessed with the model. Thus, I think that the evolutionary claims either need to be removed, or the manuscript and model need to be presented in a more thorough way.

Indeed, there are no direct mechanisms included in the model that simulate mutations and evolutionary processes. Potential influences on the evolution of bacterial species are made based on the differential growth rates depending on the spatial location and available nutrients. The enormous disparity in growth rates is likely to play a role in population development and evolution through mutation rates, natural selection and genetic drift (and the maintenance of “relic” traits in cold spots). However, as we do not simulate these processes explicitly, we have removed the terminology of evolution from the manuscript and relate to the differential population dynamics depending on the location of bacterial cells or sub-populations. We have added an additional paragraph describing a potential experimental avenue to resolve intra-sample generation time distributions that would in addition link the emerging population dynamics with evolutionary consequences.

--The paper attempts to make fairly wide-reaching conclusion about the lifespan of cells, but only in soils. Are the findings only relevant to soils? If so, why? Some of the features that seem to be implicated in longevity relate to spatial heterogeneity, but this is characteristic of many microbial habitats. What about other taxa? It would be useful if the paper would more explicitly consider the generality (or lack of) of the models and inferences that are made from them.

A key mechanism that shapes the distribution of bacterial cell ages in soil is the dynamic fragmentation of the aqueous phase combined with patchy resources in which nutrient supply is controlled by connectedness of thin water films. Other microbial habitats that are dominated by sessile communities such as sediments, marine particles or biofilms may include different mechanisms that lead to diminishing nutrient fluxes, however making inferences about the age distribution in these habitats based on our computational observations would be speculative. Concerning other taxa, bacterial motility is restricted in unsaturated soils due to the thin water films that prevents mixing of the population. This results in proliferation of a small fraction of cells that are in close proximity to the nutrient source whilst cells further away rely on diminishing nutrient fluxes that only permits negligible growth. Thus, taxa that are governed by similar processes concerning nutrient acquisition and motility would share a similar age distribution as our simulated population.

--It is well known that water content in soils fluctuates through time. While the frequency may differ according to geographic region (e.g., desert vs. temperate grassland), it’s reasonable to assume that water content varies through time, which may “erase”, disrupt, or reset the power law relationship when cold sites become hot sites, thus preventing the accumulation of old-aged individuals. Such features seem to be already be incorporated into the model, but instead, the paper seems to focus on static conditions held at different water potential. I think discussion and perhaps additional simulations might be warranted here.

This is of course true and important, however, there is an inherent separation of time scales between the connected and fragmented state of the soil aqueous phase. Considering for example a soil in a temperate region, even with 140 rainy days (this is the number for Zurich in Switzerland) a soil may remain saturated for only a few hours before it drains under the influence of gravity. In other words, the “erasing” events are short lived (typically less than 5% of the time even in temperate regions) relative to the prolonged fragmented state. Clearly these are important events for colonization of new surfaces, for convective transport of cells (and nutrients) – however, the fragmented state governs microbial life in soil (which also makes it a unique and diverse habitat relative to other compartments of the biosphere).

--The results section of the paper attempts to provide mechanistic explanations for the power law distribution of generation times by invoking the contributions of specific physical forces and physiological limits. Perhaps it makes sense not to monkey around with soil physical properties, but there’s a lot of variation in traits of microbes in real soils. For example, many bacteria in soil are facultatively anaerobic, and many populations exhibit variation in motility. If strong claims are to be made about how oxygen and motility lead to the emergence of power law distributions in lifespan, wouldn’t it be reasonable to test this with simulations where these traits are directly manipulated/controlled?

The reviewer is correct, and indeed soil bacteria are amongst the most diverse communities on this planet, trying to systematically capture variations in bacterial traits in our simulations in nigh impossible. Our primary aim was to better understand how the soil physical processes – the structure of the pore space, aqueous phase configuration, habitat fragmentation due to thin water films and resulting nutrient landscape – govern the proliferation of bacterial cells within soil hotspots. Using an obligate aerobic species, we observe the distribution in bacterial cell ages for a species that in theory can divide every 30 minutes, but is restricted due to limiting nutrient fluxes. In our simulations, facultative anaerobic growth would only be beneficial at -0 kPa and -1 kPa where the whole domain remains saturated and oxygen diffusion is limited. We recognize the limitation imposed by the choice of an obligate aerobic species and included a paragraph in the discussion section to hypothesize how facultative anaerobic species and obligate anaerobes may change the generation time distributions in saturated conditions.

Line 360: *Evidently, microbial life in soil is characterized by a vast diversity in traits and the ability to grow in absence of oxygen. Around soil hotspots with oxygen and carbon counter-gradients, the bacterial community may self-segregate into sub-populations following their oxygen preference*^1^ *and engage in cross-feeding of intermediate metabolites as demonstrated in silico*^2^ *and experimentally in bacterial colonies growing on agar plates*^3^ *or microcolonies within microfluidic devices*^4^*. In comparison to our simulations using an obligate aerobic species in saturated conditions, this scenario would lead to more rapid proliferation of anaerobically growing cells in close proximity of the carbon source whereas obligate aerobic species are restricted to leaking intermediate carbon sources with limited prospects for rapid growth. Based on these results, we would expect a shift of the generation time distribution in saturated conditions converging towards the distribution observed in intermediate hydration conditions with a greater abundance of rapidly growing cells and a slightly more pronounced tail of the distribution due to aerobic cells growing on intermediate carbon sources.*

--In the discussion, the paper talks about “unbounded range of bacterial ages”. The paper asks what the age of a bacterial cell might be. The answer provided is “practically any age”, suggesting there is no limit. Perhaps this comes from a strict interpretation of the power law based on simulations corresponding to on 30 days. But there are good reasons to believe that there are real constraints on cell age, particularly in soils, where microbes commonly experience stress (oxidative, nutritional, radiation, grazing) in their characteristically dynamic environment.

In the discussion, we specifically emphasize biotic and abiotic conditions that act to “erode” the tail of the generation time distributions. In addition, we rephrased the conclusion that the age may be “practically any age” to state that calculating mean cell ages for soil bacteria is far more complicated compared to e.g. mammalian populations. Throughout the manuscript, we have also changed the terminology to primarily talk about heavy-tailed generation time distributions (that in drier conditions may resemble a power law distribution), emphasized the role of other constraints and rejuvenation events that act to erode the tails of the distribution.

Line 414: *The original aim of estimating bacterial cell age and generation time distributions in soils in relation to heterogeneity and hydration conditions began with a very simple question: “What is the average age of a soil bacterial cell?”. In contrast to macrobiota with a finite life span where an average age in an ecosystem is well defined, providing a similar answer for prokaryotes living within heterogeneous soil is far more complicated.*

Minor comments:

--Overall, I think the paper would benefit from some polishing of the text. The writing was sometimes hard to follow. Some examples include incomplete sentences (e.g., lines 29-31). In other places, the paper uses terminology that was confusing. For example, “coexisting” (line 37) conjures up processes and theory relating to diversity maintenance, which I don’t see as being relevant given my understanding of the model structure. Similarly, there is discussion about the “emergence of relic traits”, which again seems to be outside the scope of what was represented in the IBMs. It would be advisable for any future versions of the paper to be more careful with such language.

We have modified the language of the manuscript to avoid hinting at specific theories through terminology. Specifically, we removed any speculations of evolutionary processes and relic traits. We polished the manuscript to increase readability.

--The Figure 2 caption could be improved for clarity. The methods section does a good job of explaining, but I could not understand in a “stand alone” way what was going on in panel b.

We updated the description of panel b in Figure 2 to provide more information of the simulation structure.

Line 128: *b) The model imitates a soil bacterial hotspot with a central carbon source and peripheral oxygen. Bacterial cells are represented as individual agents inhabiting an angular pore network with varying pore sizes. Diffusion of aqueous substrates and cell dispersal is dictated by thin water films within the angular pores, resulting in a patchy nutrient landscape and localized growth conditions.*

--In Figure 3 panel c, it does not seem like the power law models does a good job of fitting the simulated data for wet soil. Given this, I think it would be appropriate to conduct a more formal analysis. In other words, does a power law do a good job of describing the distribution of generation times for all moisture conditions? Should other models be considered in some cases? This is addressed to some degree in the supplement, but is less obvious in the discussion and figures in the main text.

We have added the exponentially truncated power law distribution to Figure 3 and added maximum likelihood estimates of other heavy-tailed distributions to representations of both the original and extrapolated data in the supplementary Figure 1.

--Could the authors expand their discussion with regard to how the model predictions could be explicitly tested in an experimental system, or better yet in the real world? Some approaches are alluded to in the introduction, but it seems like it would be appropriate to help guide readers towards ways of evaluating.

We added a paragraph to the discussion of the manuscript describing an experimental system that can track individual lineages within mixed populations. This system combined with whole genome sequencing may also provide insights into the missing link between bacterial generation time distributions, associated reproductive success and evolutionary forces acting on the population.

Line 404: *To resolve the bacterial age and generation time distribution of small soil volumes, novel experimental approaches are required that manage to capture the breadth of bacterial growth rates. A promising avenue is to use chromosomal barcoding of bacterial species that enables direct tracking of individual lineages*^5,6^*. In this approach, a single bacterial species is tagged with a library of unique genetic barcodes that enable to track individual lineages throughout time and observe the emerging generation time distribution. Furthermore, by combining this approach with whole genome sequencing, the influence of individual mutations on the population composition and overall evolution of the initially isogenic population can be resolved*^7,8^ *and sheds light onto the missing link between individual bacterial population dynamics and their generation time distributions with the overall population evolution.*

Reviewer #2

Review Summary

In this paper the authors generate and study the distribution of generation times in communities of bacterial species in local patches of soil. To this end, they use a previously-published spatially-explicit simulation model (Borer et al., 2019) that emulates networks of micropores and the hydrodynamics therein that govern the physical transport and exchange of gases, nutrients and microbial cells, as well as microbial metabolite exchanges and population growth. They find that the simulations yield relatively long-tailed (but not power-law) distributions of generation times across isogenic species populations, with the specific shape varying with hydration level (drier = more power-law like). The authors derive a stochastic (mixture) model to support their hypothesis that real-world bacterial generation time distributions are likely to follow power-laws, and suggest that the disparity between this analytic model and the simulations is driven by the greater realism of the simulation model.

We found the line of investigation of this study very interesting, and the manuscript is well written and easy to follow. However, we feel that there is a lot going on “under the hood” that needs clarification. In particular, the simulation results here rely heavily on the previously-published model (Borer et al., 2019) and its specific parameterisations, forcing the reader to read that paper which still leaves a few key questions unanswered (more details below). We also think that the empirical data aggregation from the literature not as comprehensive as it could be, and have some concerns about the analytic derivation of the power-law distribution of generation times.

Overall, we think that this paper uncovers something of value in terms of the relative effects of hydration regimes on generation time distributions at small/local spatial scales. But in terms of absolute effects and generation time distributions in the “real” world, we find the key claims tenuous for the reasons detailed below. If the authors instead focus on addressing the issue of hot/cold spots themselves changing over time, and restrict themselves to discussing the implications of generation time differences that their model actually resolves (i.e., in the order of tens of days), then this paper would still be a valuable contribution (with the necessary caveats about the model’s limitations). This would still allow them to engage in the discussion of side-by-side coexistence of slow generation-time microbes acting as “keepers of traits” and fast generation-time microbes undergoing rapid evolution at their maximal growth rate.

Our specific comments follow.

Major comments

The simulation model.

There very little explanation of the (previously-published) model (Borer et al., 2019) used to generate the results of this paper. This makes it hard to understand the mechanistic basis as well as robustness (in the sense of parametrisation-dependence) of the presented results, and assess what impact the assumptions inherent in the underlying model could have on the final results. Four key questions in this regard that arise for us are:

Motility: how dependent are the results on the choice of the motility model and parameterizations? For example, the parameter definitions given in Table S1 (Lines 561–563) suggest that the mode of microbial motility considered is run and tumble movement + chemotaxis, something that is mentioned nowhere else in this manuscript, and ultimately one has to go back to the previous paper, and upon reading that, one is still not sure about the robustness of the specific parameterizations chosen.

We thank the reviewers for highlighting the omission of describing the motile behavior of the cells. In previous simulations, we investigated the influence of bacterial motility on the resulting spatial organization of the cells, both by changing the tumbling probability and/or the cell motility. Overall, motility of the bacterial cells is dictated by the water film thickness of the angular pores that results in a fragmentation of the habitat. Even in saturated conditions (where bacterial motility is not influenced by the pore geometry), changes to the parameters (chemotactic sensitivity and/or swimming velocity) did not significantly affect the spatial distribution and total population size as the number of simulated agents homogenizes the influence of any single cell.

There is no mention of species interactions in the local domains. The rGEM-based modelling of microbial cell growth and division presumably yields interactions between (and within) microbial species, but this issue is not addressed at all in the paper. It may well be that interactions are moot here, but its unclear currently.

For these simulations, we restricted the bacterial population to a single species where growth is represented using Monod kinetics with limitation terms for oxygen and carbon that enables a minimum division time of 30 minutes. We have chosen this simplest of cases to highlight the influence soil physical properties have on the bacterial population dynamics and demography whilst omitting additional factors such as different bacterial traits (facultative anaerobic or anaerobic growth, bacterial dormancy, osmotic bursting or grazing) that would influence the shape of the bacterial cell age distribution. We have added a paragraph in the discussion hypothesizing how the generation time distribution may be affected when considering facultative/obligate anaerobic species and trophic interactions.

Line 360: *Evidently, microbial life in soil is characterized by a vast diversity in traits and the ability to grow in absence of oxygen. Around soil hotspots with oxygen and carbon counter-gradients, the bacterial community may self-segregate into sub-populations following their oxygen preference*^1^ *and engage in cross-feeding of intermediate metabolites as demonstrated in silico*^2^ *and experimentally in bacterial colonies growing on agar plates*^3^ *or microcolonies within microfluidic devices*^4^*. In comparison to our simulations using an obligate aerobic species in saturated conditions, this scenario would lead to more rapid proliferation of anaerobically growing cells in close proximity of the carbon source whereas obligate aerobic species are restricted to leaking intermediate carbon sources with limited prospects for rapid growth. Based on these results, we would expect a shift of the generation time distribution in saturated conditions converging towards the distribution observed in intermediate hydration conditions with a greater abundance of rapidly growing cells and a slightly more pronounced tail of the distribution due to aerobic cells growing on intermediate carbon sources.*

How many species / isogenic strains were introduced into a patch? Were the 1000 cells “inoculated homogeneously across the domain” per species, or across all species? Are extinctions possible in this system, and how are they handled?

The simulations consider the inoculation of 1000 individual cells, where each cell is represented using the exact same parameters (i.e. a single isogenic species). The inoculated cells are distributed randomly across the whole domain. Thus, the bacterial species itself does not go extinct but individual cell lineages (tracked as the original inoculated cell and all of its offspring) may go extinct. We clarified the inoculation of the cells in the methods section.

Line 448: *A total of 1000 bacterial cells are inoculated homogeneously across the domain (representing an isogenic population where each cell has the same growth parameterization) with a total simulation time of 30 days at 10 s time steps.*

As the focus here is on complex communities, is the model able to produce any elements of realistic macroecological patterns (e.g., a “canonical” species abundance distribution) seen in (soil or otherwise) microbial communities (Grilli, 2020)? Maybe this last point is a bit demanding because the published model has been validated to some degree in the previous paper, but these are complex dynamical systems, and some consideration/discussion of this is warranted.

This response if somewhat similar as our response above, emphasizing that we have not considered highly complex communities but rather opted for the simplest bacterial population of a single obligate aerobic species to observe the emerging age distribution due to hydration conditions and related nutrient landscapes (additional complications would limit interpretation and would be difficult to disentangle driving factors). Due to these simplifications, we cannot make predictions of realistic species abundance distributions in soil based on our mechanistic framework.

The empirical data and associated issues.

We agree that practically no studies have measured community-level generation times including variation within isogenic (species) lineage level variation. However, in our view these data can still be used to partially test the simulated model’s predictions. Specifically, the central tendencies (means or medians) of the distributions underlying a mixture distribution should follow some canonical distribution (possibly also a power-law in this case?). Therefore, it would be meaningful to compare the distribution of means or medians of species-specific generation times emerging from model-simulated data with those of real datasets. With reference to this, the authors should consider published datasets on maximum growth rates that include soil- derived taxa (Corkrey et al., 2016; Smith et al., 2019). These also account for temperature variation, which would allow for a more thorough analysis of the (temperature-corrected) distributions of generation times of potential, randomly constituted local bacterial communities. That is, this would offer a null or neutral model for what the generation time distribution of mean/median values in multi-species communities might look like. This may also lead to the derivation of the generation time distribution of a local, well mixed microbial community under idealized nutrient and substrate conditions, providing a theoretical benchmark to compare more realistically mixed communities against.

In terms of the plot shown in Fig 1, the empirical data panel is not a relevant point of comparison to the panel based on the simulations below it, because as the authors themselves point out, the literature reports only mean values of single populations. It would therefore be helpful to show another plot of (a representative) distribution of population means of simulated data so that a direct comparison can be made with empirical data.

Indeed, comparing the literature values with the distribution of bacterial generation time distributions of a single population is not a valid comparison. In order to disentangle this, we split Figure 1 to show the means of the simulated population (that is more comparable to the literature data) and a separate panel which shows the distribution of the generation times for the population in the simulations.

Also, it is not clear if/how the empirical data has been selected to generate a representative sample of generation times (the data aggregation are not described at all in the methods or elsewhere). For example, what sort of method is the 4-year data point based on? Is it really comparable to the others?

A major limitation that a study of this type faces is lack of definitive data across long time scales. We pretty much followed the reviewer’s line of reasoning expressed above “*these data can still be used to partially test the simulated model’s predictions”* – in other words, we are forced to use imperfect and perhaps not even fully comparable data sets to test the model with the caveats that we list in the revised manuscript.

To provide more information on the assimilated literature data, we assembled the values, methods and samples in the supplementary Table 1 and added a cross-reference to the Figure legend.

Line 86: *a) Comparison of literature values*^9–16^ *and mean generations times in the simulations for different hydration conditions. Further information on the soil samples and quantification techniques of the literature values can be found in Table S1.*

And although the authors suggest that empirical data are biased towards detecting younger cells, why are the generation times in the empirical data in Fig 1 actually much longer than the simulated data?

When comparing the experimental values of mean generation lengths compared to the distribution of generation times around a hotspot, the experiments indeed seem to have longer average generation times. As shown in the newly added mean simulation generation times, these already shift towards longer generation times. Finally, the experiments typically measure growth rates in larger soil volumes with multiple bacterial hotspots but also a far greater volume of inactive soil. In comparison, our simulations observe the generation time distribution in proximity of a bacterial hotspot with a constant carbon source at relatively high concentration (0.1 mM). These conditions enable rapid growth in close proximity of the source for the majority of hydration conditions (excluding saturated pore networks) that shift the distribution towards shorter generation times.

Analytic derivation of the generation times distribution.

The relevance of these results to the question of microbial age distributions hinges on the discovery of a power law scaling of generation time, which suggests a finite probability of observing extremely large times between cell division events. The derived exponent (α = 2) differs substantially from the range of exponents found across matric potentials (α = 1.2 1.35). This deviation is mentioned briefly in the discussion (Lines 250–255), but further investigation is deferred to future studies. This is matched by a derivation that is short and lacking in detail (Lines 375–392). We think that clarifying the assumptions made in the steps of this derivation is necessary to make the results of this paper clearly understandable.

When revising the manuscript, we have changed the focus on providing visual cues on how different heavy-tailed distributions fit to the simulation data but avoided to compare the specific parameters of each of them. Primarily, although different conditions may still give rise to a power law distribution of cell ages, these would only be valid for a certain range in real soil and we focused efforts to understand how different regimes may act on eroding parts of the distribution. We expanded the derivation of the heuristic model and added all of the underlying assumption to the results section of the manuscript. We derived the model in the case where the generation time distribution follows a Gamma distribution and numerically integrated the equation to estimate the influence of different growth rate distributions on the emerging cell age distributions.

Beyond this, the derivation appears to implicitly make the assumption (Eq. 3) that the distribution of growth rates (k) can be treated to be uniform, and so the integral can be performed without a weighting function. In the supplementary text to (Şimşek and Kim, 2019) (cited in the manuscript) the impact of non-uniform distributions of growth rates is considered. We wonder if this can be used to explain either the substantial deviation of the exponents or the fact that exponentially truncated power laws appear to fit better than power laws in the majority of cases.

This is a great point and, in the revision, we included an analysis of how different weights change the resulting cell age distribution. Simsek and Kim applied a nonhomogeneous weight function before integration that is simple enough to find an analytical derivation. They chose an exponential distribution $h\left( k \right)=ae^{-ak}$ and $h\left( k \right)=\frac{2a}{b^{2}}*\left( b-ak \right)$, both of which converge towards homogeneous distributions at small values a. This result in a deviation from the power law at smaller values, whilst retaining a power law behavior in the tail of the distribution. Translated to our study, their choice of weight function would favor extremely slow growing bacterial cells and reduce the abundance of rapid growth conditions. To explore more nuanced weight functions, we use weight functions derived from log normal distributions and use numerical integration to derive the resulting community wide cell age distributions. In contrast to Simsek and Kim, our choice of weight distributions predominantly act to erode the tail of the power law distribution since extremely slow growth is less represented. We added Figure 5 to the manuscript to discuss the influence of different parameter choice of the coefficient of variance, our choice of weight distributions and resulting community cell age distributions.

There is an additional issue: the time-scale of physical mixing of bacterial cells, nutrients, and carbon substrates is annual or shorter (through periodic or intermittent precipitation and inundation). That is, arguably, mixing happens frequently enough to make cold and hot spots ephemeral physical entities in the timescales relevant here. Therefore, the wet/dry patches that the authors define (corresponding to population-dynamically hot/cold spots) are not static, but must surely themselves evolve over, say, a period weeks or months at the longest. With these considerations, any shortcomings of the model apart, we feel that the claim that cells and lineages of the (extreme) age suggested by this work seem highly unlikely empirically, unless one is talking about much deeper zones in soil than what is the focus of the modelling. Overall then, although the possibility of ancient lineages co-existing with younger ones is an intriguing one, the results of the current paper are far from convincing in that regard. Ultimately, fits to the power-law are weak, and the exponential declines across all regimes (including the driest ones) not given due regard. We are unable to judge how valid the method of extrapolating the distributions of generation times mentioned in lines 154–157 is.

We fully agree with the reviewers and have added all assumptions concerning Equation 1 to the manuscript and state that these provide an important upper boundary for the interpretation of the results. When observing the data, the numerically integrated model shows a heavy erosion of the power law distributed cell ages and the probability of a cell having an age of a month becomes vanishingly small. To drive this point home that the heavy-tailed distributions need to be interpreted under consideration of the limiting assumptions, we have added the assumption of the underlying equations used for the derivation of the heuristic model to the results section and added a “statement of caution” to the discussion concerning the interpretation of its results.

Line 383: However, we emphasize that results of the heuristic model need to be interpreted in light of the assumptions of the underlying equations outlined in the results section.

Other comments

What about the role of (across-species) scaling of metabolic rate with cell size in generating power law distributions of generation times (DeLong et al., 2010)? Size presumably plays a key role in motility as well. Maybe worth discussing as it provide some additional mechanistic understanding about the real world phenomenon even if size is not considered in the simulation model.

Within the model, the increase in biomass per cell is proportionate to the cell mass and increases following an explicit Euler scheme (an exponential model). This essentially is equivalent with the metabolic rate increasing with cell mass (and thus volume), i.e. if a cell is double the size of another it can produce double the biomass in the current time step compared to the latter when experiencing the same nutrient conditions. This process of heavier cells producing more biomass has been verified experimentally at single cell resolution using the buoyant mass of microbial species^17^. The effect of different cell sizes on motility is not included directly in the model. The cell swimming velocity is a function of the water film thickness^18^ within the pores and is restricted when the water film thickness is smaller than the bacterial cell size at division. Thus, the variation in cell size is not translated into the motility of the cells, but is captured in the growth characteristics of the individual cells and lineages within the population.

Lines 12–14: The claims made early in the abstract seem too bold to be supported by the present results of the paper or the literature. Specifically, the claim in the abstract that “much older living microorganisms (than 4850 years) are likely to be found in most soils” does not appear to be supported by any literature cited in the paper.

We have modified the abstract and removed this claim and focus on the concurrent existence of young and old bacterial cells within the same volumes.

Line 11: *Resource patchiness and aqueous phase fragmentation in soil may induce large differences local growth conditions at submillimeter scales. These are translated to vast differences in bacterial age from cells dividing every thirty minutes in close proximity to plant roots to very old cells experiencing negligible growth in adjacent nutrient poor patches.*

Lines 20–23: This part of the abstract is a bit vague and makes no mention of the specific mechanisms investigated.

We have rephrased the sentence to introduce processes discussed later in the manuscript in more detail.

Line 19: *The results imply that bacterial strains with beneficial mutations may exist concurrently with their ancient lineages that have not been selected out of the population due to local variations of growth rates and spatial fragmentation within small soil volumes – a feature unique to microbial life. Our study highlights a potential mechanism for the remarkable breadth of soil bacterial ages and the emergence of a soil genetic reservoir critical for ecosystem stability and function.*

Lines 29–31: Something is wrong with the sentence here (appears to be a fragment).

We have changed the sentence to increase readability.

Line 43: What are these relic traits? And example or two would help.

We have removed the terminology of relic traits from the manuscript during revision of the manuscript.

Lines 103–111: Hydrological regime is clearly key to the emergent patterns from the simulations, but only gets mentioned at the start of the Results section rather than in the Introduction (or Abstract). Also, much of this start of the Results seems like information that should be presented in the Methods rather than here.

We have moved the description of how the hydrological domain influences microbial life to the introduction and any description of the mathematical model to the methods section.

Line 48: *Soil hydration status has been shown to be a key variable that governs multiple functions of microbial life such as community structure*^19^*, horizontal gene transfer rate*^20^*, cell dispersal*^21–23^ *and nutrient diffusion (both aqueous and gaseous)*^24^*. From a nutrient flux perspective, too wet or too dry soil conditions are generally unfavorable for bacterial activity, due to either a lack of oxygen when saturated or limited aqueous nutrient diffusion in dry soil. Optimal nutrient and gaseous fluxes, and related high growth rates, are often supported at intermediate hydration levels*^25^*.*

Lines 113–114: The results pertain specifically to obligately aerobic bacteria. This is briefly defended in the Methods section. Some discussion of how these results be effected if e.g., dominantly, or facultatively anaerobic bacteria were considered?

We added a paragraph in the discussion to explore how facultative and obligate anaerobic species may change the generation time distribution.

Line 126: There isn’t really enough information provided in these plots to assess how well the power- law fits the datasets. The details are in the Supp info, but the fact that the data pretty much never fit a strict power-law would be better off shown in the main text.

We have shifted the terminology in the manuscript to heavy-tailed distributions and added visualizations to other heavy-tailed distributions in the supplementary information for the reader to see how these distributions look in relation to the observed data.

Lines 183–184: Exactly what is meant by “Power law distributed cell ages induce a broad range of reproductive success” could be clarified a bit.

We have rephrased the sentence to increase clarity.

Line 200: *A heavy-tailed generation time distribution suggests a broad range of reproductive success where a few lineages dominate the total bacterial biomass due to rapid proliferation whereas lineages with long generation times contribute little to the total biomass of a population.*

Lines 189–190: The method used for the classification of lineages is fairly opaque – please ex- plain/clarify further

We have included a more precise description of a lineage in the manuscript.

Line 202: *In this context, a cell lineage is defined as the inoculated cell at the simulation initialization and all of its progeny with the reproductive success being the maximum generation achieved by a single lineage during the simulated time. Lineages with high reproductive success are those very few inoculated cells and their progeny that contribute disproportionally to the final population biomass, and are further defined as the dominant fraction.*

Lines 253–254: Reference is made to an “assumption-free mechanistic model”, a model without assumptions ceases to be a model, in our opinion.

We agree with the reviewers and removed this statement when rewriting this section of the discussion.

Lines 289–303: A large number of possible countervailing mechanisms are mentioned here, and it’s noted that the actual generation time distribution will be a balance between the mechanism studied in this manuscript and these counteracting mechanisms. Given the importance placed on these here and the manuscript’s stated aim of determining the possible ages of soil microbes is that no way that the impact of counteracting mechanisms could have been considered even briefly in the Results?

Including all potential mechanisms in the model is nigh impossible and making a specific selection of a few mechanisms may bias the results towards a specific shift in the generation time distribution. We have thus resorted to only look at the influence of hydration conditions on the spatial nutrient landscape and emerging bacterial growth rates that are balanced by a maintenance rate (essentially the only mechanism for bacterial cell death in the model). We have restricted the ourselves to discussing these processes but would be excited about future models and/or experiments that could provide insights into the relevant importance of such processes.

Line 305: Why is evolutionary rate a function of nutrient conditions? Is this entirely due to faster growth rate meaning more cell divisions and thus more opportunity for mutations?

Indeed, the assumption is was that (neglecting horizontal gene transfer from other species within the domain), genetic variation within the population emerges from beneficial mutations during DNA replication and is thus a function of inter division times/growth rates that can be linked to nutrient conditions. As we have removed speculations of potential evolutionary forces, we have also rephrased this sentence.

Lines 331–333: The conclusion that microbes can be practically any age seems too bold a conclusion given the current results.

We have changed the concluding remarks of the manuscript and stated that elucidation of the mean bacterial cell age is more complicated when compared to mammalian populations.

Line 414: *The original aim of estimating bacterial cell age and generation time distributions in soils in relation to heterogeneity and hydration conditions began with a very simple question: “What is the average age of a soil bacterial cell?”. In contrast to macrobiota with a finite life span where an average age in an ecosystem is well defined, providing a similar answer for prokaryotes living within heterogeneous soil is far more complicated.*

Eqn 1: Worth mentioning that this is for symmetric division and defending that assumption.

We have added the assumptions of Equation 1 to the results section.

Lines 531–538: The choice of line style and color makes the lines in Fig S1A and S2A hard to see.

We have changed the color scheme in the new SI Figure 1 to hopefully increase visibility of the different distributions.

Fig S1, Line 535: a negative sign is missing before “3 kPa”

We thank the reviewers for the attention to the Figure and changed it in the new SI Figure 1.

Lines 552–559: A lot is expressed in this figure caption, whilst the mention of this figure in the main text is very brief. Some of the explanation given here would be more helpful if shifted to the main text.

We have added a lot of details how hydration conditions shape bacterial motility to the introduction where we generally introduce how hydration conditions shape microbial life in soil.

Line 54: Hydration conditions also dictate bacterial dispersal, where water saturated conditions often facilitate convection or motility through the pore network thus enabling relocation over large distances and introduction of bacterial cells to more favorable locations. In contrast, drier conditions and associated thin water films restrict bacterial cell motility and result in a fragmented habitat where pinned bacterial cells are restricted to diminishing diffusive nutrient fluxes and limited prospects for proliferation.

References:

References:

1.	Borer, B., Tecon, R. & Or, D. Spatial organization of bacterial populations in response to oxygen and carbon counter-gradients in pore networks. Nature Communications 9, 1–11 (2018).

2.	Borer, B., Ataman, M., Hatzimanikatis, V. & Or, D. Modeling metabolic networks of individual bacterial agents in heterogeneous and dynamic soil habitats (IndiMeSH). PLOS Computational Biology 15, e1007127 (2019).

3.	Cole, J. A., Kohler, L., Hedhli, J. & Luthey-Schulten, Z. Spatially-resolved metabolic cooperativity within dense bacterial colonies. BMC Systems Biology 9, 1–17 (2015).

4.	Dal Co, A., Ackermann, M. & van Vliet, S. Metabolic activity affects the response of single cells to a nutrient switch in structured populations. Journal of The Royal Society Interface 16, (2019).

5.	Blundell, J. R. & Levy, S. F. Beyond genome sequencing: Lineage tracking with barcodes to study the dynamics of evolution, infection, and cancer. Genomics 104, 417–430 (2014).

6.	Jahn, L. J. et al. Chromosomal barcoding as a tool for multiplexed phenotypic characterization of laboratory evolved lineages. Sci Rep 8, 6961 (2018).

7.	Hegreness, M., Shoresh, N., Hartl, D. & Kishony, R. An equivalence principle for the incorporation of favorable mutations in asexual populations. Science 311, 1615–1617 (2006).

8.	Levy, S. F. et al. Quantitative evolutionary dynamics using high-resolution lineage tracking. Nature 519, 181–186 (2015).

9.	Barber, D. A. & Lynch, J. M. Microbial growth in the rhizosphere. Soil Biology and Biochemistry 9, 305–308 (1977).

10.	Söderberg, K. H. & Bååth, E. Bacterial activity along a young barley root measured by the thymidine and leucine incorporation techniques. Soil Biology and Biochemistry 30, 1259–1268 (1998).

11.	Bååth, E. & Johansson, T. Measurement of bacterial growth rates on the rhizoplane using 3H-thymidine incorporation into DNA. Plant and Soil 126, 133–139 (1990).

12.	Olsson, S., Bååth, E. & Söderström, B. Growth of Verticillium dahliae Kleb. hyphae and of bacteria along the roots of rape (Brassica napus L.) seedlings. Canadian Journal of Microbiology 33, 916–919 (1987).

13.	Bowen, G. D. & Rovira, A. D. Microbial colonization of plant roots. Annual Review of Phytopathology 14, 121–144 (1976).

14.	Bennett, R. A. & Lynch, J. M. Bacterial growth and development in the rhizosphere ofgnotobiotic cereal plants. Microbiology 125, 95–102 (1981).

15.	Christensen, H., Funck-Jensen, D. & Kjøller, A. Growth rate of rhizosphere bacteria measured directly by the tritiated thymidine incorporation technique. Soil Biology and Biochemistry 21, 113–117 (1989).

16.	Bloem, J., de Ruiter, P. C., Koopman, G. J., Lebbink, G. & Brussaard, L. Microbial numbers and activity in dried and rewetted arable soil under integrated and conventional management. Soil Biology and Biochemistry 27, 655–665 (1992).

17.	Godin, M. et al. Using buoyant mass to measure the growth of single cells. Nat Methods 7, 387–390 (2010).

18.	Wang, G. & Or, D. Aqueous films limit bacterial cell motility and colony expansion on partially saturated rough surfaces. Environmental Microbiology 12, 1363–1373 (2010).

19.	Kleyer, H., Tecon, R. & Or, D. Rapid shifts in bacterial community assembly under static and dynamic hydration conditions in porous media. Applied and environmental microbiology 86, (2019).

20.	Tecon, R., Ebrahimi, A., Kleyer, H., Levi, S. E. & Or, D. Cell-to-cell bacterial interactions promoted by drier conditions on soil surfaces. Proceedings of the National Academy of Sciences of the United States of America 115, 9791–9796 (2018).

21.	Dechesne, A., Wang, G., Gulez, G., Or, D. & Smets, B. F. Hydration-controlled bacterial motility and dispersal on surfaces. Proceedings of the National Academy of Sciences 107, 14369–14372 (2010).

22.	Ebrahimi, A. N. & Or, D. Microbial dispersal in unsaturated porous media: Characteristics of motile bacterial cell motions in unsaturated angular pore networks. Water Resources Research 50, 7406–7429 (2014).

23.	Tecon, R. & Or, D. Bacterial flagellar motility on hydrated rough surfaces controlled by aqueous film thickness and connectedness. Scientific Reports 6, 19409 (2016).

24.	Tecon, R. & Or, D. Biophysical processes supporting the diversity of microbial life in soil. FEMS Microbiology Reviews 41, 599–623 (2017).

25.	Ebrahimi, A. & Or, D. Hydration and diffusion processes shape microbial community organization and function in model soil aggregates. Water Resources Research 51, 9804–9827 (2015).
