# Supplementary material for: Bacterial age distribution in soil – Generational gaps in adjacent hot and cold spots
Source: PLoS Comput Biol. 2022 Feb 25;18(2):e1009857. doi: 10.1371/journal.pcbi.1009857 (PMC8906644; doi:10.1371/journal.pcbi.1009857)
Supplement: Attachment — Submitted filename: Responses to Reviewer_age_distributio.docx [file pcbi.1009857.s013.docx]

Responses to Reviewer’s Questions

First, we genuinely appreciate the additional input by the reviewers that inspired us to make changes leading a much-improved manuscript. We have conducted a series of new simulations considering non-motile bacterial cells, oxygen independent bacterial species and complex dynamic simulations of soil environment subjected to periodic rainfall events that temporarily alter the aqueous phase in the simulated domain and allow connectivity and higher levels of diffusion and cell dispersion. We added these new results as supplementary information and cross-referenced the new material in the revised discussion. We further changed the language to avoid mention of evolutionary processes in the abstract and author summary. Finally, we mention the important link between cell-size variation and metabolic rate in the discussion. Again, we would like to thank the reviewers for their time in assessing the manuscript and providing great suggestions to improve our work.

Reviewer #1: Overview: This is an evaluation of revised manuscript that I made comments on previously. In general, it seems like the authors have made attempts to correct some of the major issues that were identified, but have elected not to explore some of the other topics that were raised by both reviewers. So, perhaps I’m left a little bit less excited about the paper, but as stated previously, I still think the paper is valuable in that the simulations generate some interesting patterns that could perhaps be experimentally tested.

1. As noted before by both reviewers, the abstract makes some bold claims. The authors state that they have toned things down, but there still seems to be some disconnect. Also, one of my major concerns about the paper was that it was inappropriately making reference to evolution. The authors indicated that they eliminated this focus, but then, in the abstract, the paper is talking about the role of beneficial mutations. Why? This is not within the scope of the paper based on what the model does. While it may be reasonable to talk about how future versions of the model could explore the evolution of lineages, it is not appropriate in the current model which is made up of isogenic, non-mutable individuals in the absence of selection. At the end of the abstract, there’s reference to the emergence of a genetic reservoir. Again, there’s no genetic variation among the individuals. I’m a bit puzzled and feeling misled.

The reviewer is right that model simulations do not include genetic variations or reflect on long term evolutionary processes other than (possibly) the potential of vertical gene transfer observed by proliferation of individual lineages (we certainly did not intend to mislead readers concerning this aspect). In our second revision, we further removed the mentioning of mutations and genetic variation from the abstract and highlighted that our model does not include evolutionary aspects and that the discussion of this topic is speculative.

Lines 120 to 124: *While the numerical and analytical models are instrumental in providing insights into drivers and modifiers of bacterial population age and generation time distributions, their short time scales and numerous simplifying assumptions regarding bacterial traits, diversity and gene flow preclude their use for making inferences regarding potential evolutionary processes or shifts in population diversity, and we thus treat such potential generalizations as mere speculations.*

2. It seems that the authors have side-stepped an important issue raised by both reviewers. Soil moisture is highly variable through time and has implications for the primary pattern of the paper: that is, a heavy-tail age distribution of cells within a smaller area. I asked whether the frequent rewetting of soil would erode this pattern. The other reviewer asked about the time-scale of mixing owing to periodic precipitation (i.e., hot and cold spots change over time). The authors argue that this is not relevant because of the sufficient discrepancy in time scales between rain events and growth rates, but this doesn’t make sense to me. The authors argue that there are 140 rain days in Zurich. If rainfall is more or less equally distributed across the simulated area of habitat, this would mean that cells in soils are experiencing rewetting (and growth) every 2.6 days. This falls right within the distribution of (measured and simulated) generation times of soil bacteria (Fig. 1). Yet, simulations seem to run for ~30 days without precipitation (Fig. 2). Given this, I think it is very reasonable to ask how temporal fluctuations in soil moisture affect the heavy-tail age distribution. Are rain events in mesic ecosystems (like those in Zurich), homogenizing growth rates on times scales that make the reported heavy-tail age-distribution transient, or perhaps even trivial? Or, maybe these patterns are more important in arid ecosystems. In any case, it seems like it wouldn’t be hard to run such a simulation. I inquired about the generality of the pattern, and the authors argued that the pattern was most relevant to soils. That is fine, but if the work is meant to capture important features of soil ecosystems, then maybe it would be good to address one of its most universal aspects: soil moisture is temporally dynamic.

We agree with the reviewer that soil is highly dynamic in both time and space and the variation in soil moisture is a key driver of numerous soil biological processes. Overall, we hypothesized that episodic wetting events that temporarily enhance the influx of nutrients and extend the range of bacterial cell dispersal would not significantly alter the observed patterns of cell generation time distributions obtained under stationary hydration conditions. To test this hypothesis, we performed additional simulations that include dynamic variations in hydration conditions simulating three rain events during a thirty-day period (see the figure below which is added to the supplementary information). Overall, each rain event resulted in 6h of fully saturated soil conditions (reflecting an extreme wetting event) followed by 18h of intermediate moisture conditions due to internal drainage towards so-called field capacity state (-3 kPa) and finally settling into drier conditions (-7 kPa) until the next rain event. The initially carbon rich domain is rapidly depleted due to rapid proliferation of bacterial cells and each rain event results in a brief influx of carbon that is subsequently consumed. When comparing the resulting generation time distribution for the simulated dynamic conditions to the original static conditions, we observe a slight shift in the distribution towards shorter generation times but the overall population distribution resembles that of the average hydration conditions of the soil (in this case -7 kPa). This is due to the relocation of individual times during saturated conditions towards more favorable conditions, acting to erode the tail of the distribution. We included these observations in the discussion of the manuscript and added the figure as supplementary material.

Lines 406-410: *If for instance a rainfall event shifts the local growth rate distribution towards faster growth rates (e.g. from the red line to the green or blue in Figure 5b), the expected cell age distribution would become skewed towards younger cells for a brief period of time until water drains and soil field capacity is reached (often within a few hours) which we verified using a numerical simulation containing dynamic moisture conditions (Fig. S6).*

**Figure S6: Dynamic hydration conditions and resulting generation time distribution.** a) We simulate three rain events in a thirty-day period by including dynamic variations of the moisture content. Each rain event constitutes of 6h saturated soil conditions (-1 kPa) followed by 18h of intermediate moisture conditions during which the aqueous phase redistributes (-3 kPa) and drier conditions at the characteristic hydration condition of the soil (-7 kPa) until the next rain event. Each rain event is characterized by an influx of carbon due to the higher aqueous diffusion fluxes within saturated pores. The inset shows details of a single cycle with arbitrary units for both carbon dynamics and hydration conditions. b) Dynamic conditions result in a slight shift of the overall generation distribution towards shorter generation times. This is due to the relocation of individual times during saturated conditions towards more favorable conditions, essentially acting to erode the tail of the distribution.

3. The authors also did not seem interested in exploring variation in functional traits that underly important aspects of demography in environments with different moisture levels, namely motility and tolerance to oxygen. The authors responded that exploring variation in these (and others) is “nigh impossible”. But it seems like the models used here are the perfect tool for exploring this type of question. For example, what would the heavy-tail age distributions look like if bacteria were not obligately aerobic? Perhaps I am underestimating the amount of work it would take to address, and I’m suggesting that this needs to be done. But by electing to explore these processes, there ends up being a disconnect between the bold generalizations and what was actually done with the models.

In contrast to the reviewer’s impression, we are very keen on investigating these processes, however exploring the numerous combinations of different bacterial traits and soil physical conditions would lead to a convoluted set of results that would go well beyond the scope of the study. For instance, although parameterizations of Monod type kinetics to simulate facultative anaerobic bacteria are available (e.g. in iDynomics^1^), these usually include a whole suite of additional parameters such as the oxygen concentration at which cells switch to anaerobic metabolism, the anaerobic growth rate, anaerobic substrate affinities and lag-phase after the switch to anaerobic metabolism. Disentangling the actual shift in generation time distribution for facultative anaerobic species when compared to obligate aerobes would then be most likely a function of the choice of parameterization. Nevertheless, we have generated new simulations that explore the role of non-motile bacterial species (by setting their swimming velocity to 0), and hypothetical bacterial cells that are oxygen independent (i.e. not revert to facultative anerobic parameterization but break the dependence on the carbon-oxygen cross-gradients frequently observed in soil).

We inoculated the same number of bacterial cells initially (i.e. 1000 cells) which resulted in 1000 sparsely distributed colonies in the case of non-motile species as seen in the Figure below. These colonies were unable to self-engineer the oxygen concentration in saturated conditions, which results in a scenario where the whole population is governed by the diffusive flux of carbon to the individual colonies. For drier conditions, the higher flux of carbon from the hotspot to colonies further away (there are not enough cells in close proximity of the carbon source to intercept all of the nutrients) results in more rapid overall growth of the population compared to the simulations with motile cells. This results in a homogenization of the generation time distributions that are similar to the distribution at optimal growth conditions (-3 kPa).

**Figure S4: Influence of cell motility on bacterial proliferation and generation time distributions.** a) Spatial distribution of bacterial cells in the soil microbial hotspot domain that are clustered at the inoculation point. Considering non-motile bacterial cells, small sessile colonies form around each inoculated cell that results in high localized competition for resources. Since these colonies are sparsely distributed (i.e. overall low cell density in the domain), they do not sufficiently alter or self-engineer (i.e. consume carbon or oxygen) the environments around the hotspots, subsequently, the system is governed primarily by the diffusive flux of carbon through the aqueous phase. b) This diffusive limitation results in overall population sizes that are an order of magnitude smaller when compared to motile bacterial species. c) The diffusive limitations also induced homogenization of the generation time distribution for these species where under all hydration conditions, very similar generation time distributions were realized with the exception of saturated conditions in which the cells closest to the carbon source are additionally oxygen limited and thus have a heavier tail of the distribution.

For the oxygen independent simulations, we removed oxygen from the simulation such that bacteria are only limited by carbon. In these simulations, saturated conditions (-0 and -1 kPa) showed a far stronger proliferation of the bacterial population where the total population size is strongly correlated with the total aqueous diffusion in the domain (as shown in the Fig. S3). For all other hydration conditions, the final population size is similar (but slightly higher as oxygen limitation is negligible) when compared to the simulations with obligate aerobic species. This trend is reflected in the observed generation time distribution of the species where there is a clear shift in the distribution towards shorter generation times in the saturated condition (-1 kPa) whereas all other hydration conditions remain similar.

Figure S5: Comparison of simulation between obligate aerobic and oxygen independent (facultative anaerobic) species. a) In comparison to the obligate aerobic species, removing the oxygen limitation results in rapid proliferation of the bacterial population in saturated conditions (since they are not limited by the slow diffusion of oxygen through the saturated pores) and generally a slightly larger population size. All other terms (growth rate, maintenance and yield) were kept congruent to the obligate aerobic species for sake of comparison. b) Overall, the generation time distributions are very similar to the obligate aerobic scenario for unsaturated conditions. In saturated conditions, the rapid proliferation of the cells results in a shift of the generation time distribution towards shorter generation times (as highlighted by the arrow). However, nutrient limitation due to rapid growth of all cells (relocation of the entire population towards the carbon rich center) results in longer average generation times when compared with intermediate hydration conditions (-3 kPa).

We have added both of these insights into the discussion of the manuscript with references to the figures in the supplementary material.

Lines 362 to 367: *Motility is an important trait for soil bacterial species. In comparison to motile obligate aerobic species, non-motile bacterial species produce small colonies that are exposed to local growth conditions and unable to relocate towards more favorable conditions. Individual colonies are limited by nutrient flux through the aqueous phase and cannot self-engineer the overall nutrient landscape of the hotspot, resulting in a homogenization of the generation time distribution across hydration conditions (Fig. S4).*

Lines 369 to 375: *We further investigated how removing the oxygen dependency for growth (essentially simulating motile facultative anerobic species) changes the generation time distribution for all hydration conditions (Fig. S5). In this case, saturated conditions (-0 and -1 kPa) resulted in a vastly higher overall population size and shift in generation time distribution towards shorter generation times when compared to the obligate aerobic species simulations. For unsaturated conditions, the changes in population size and generation time distributions are minimal, confirming that the system is governed by diminishing carbon diffusive fluxes.*

Reviewer #2: The authors have made extensive revisions to address the points raised by both reviewers. Overall, we are satisfied with the revisions, except one point: We appreciate the fact that the relative tractability of a single species (but multi-lineage) model provides mechanistic insights that a more complex community model wouldn't. However, it is hard to argue that a single species can produce a generation time distribution that is comparable to that generated by multiple species in a community. This is partly because cell-size variation is greater across rather than within species in microbes (as is typically the case in other organismal groups). Now it may well be that for community of multiple lineages of a single species, spatial/environmental structure will produce a generation time distribution that is *qualitatively* the same as that generated by a multi-species community. But this cannot be determined from the current theoretical framework as such. Therefore, we encourage the authors to address this disparity (in words) in their paper's discussion.

We thank the reviewer for the positive assessment of our improvements and have included a reference to the importance of cell size variation on the metabolic rate to the manuscript. As we do not specifically investigate this mechanism with our numerical simulations, we refrain from making assumptions how the inclusion would shift the generation time distribution.

Liens 361 to 362: *For instance, cell-size variation across species plays a critical role in the metabolic rate and may thus significantly influence the generation time distribution for different cells*^2^*.*

1.	Lardon, L. A. *et al.* iDynoMiCS: Next-generation individual-based modelling of biofilms. *Environmental Microbiology* **13**, 2416–2434 (2011).

2.	DeLong, J. P., Okie, J. G., Moses, M. E., Sibly, R. M. & Brown, J. H. Shifts in metabolic scaling, production, and efficiency across major evolutionary transitions of life. *PNAS* **107**, 12941–12945 (2010).
